# Weighted correlation network and differential expression analyses identify candidate genes associated with *BRAF* gene in melanoma

**DOI:** 10.1186/s12881-019-0791-1

**Published:** 2019-03-29

**Authors:** Bin Zhao, Yanqiu You, Zheng Wan, Yunhan Ma, Yani Huo, Hongyi Liu, Yuanyuan Zhou, Wei Quan, Weibin Chen, Xiaohong Zhang, Fujun Li, Yilin Zhao

**Affiliations:** 1 School of Medicine, Xiamen University, Xiamen, Fujian China; 2 The Department of Clinical Laboratory, the Second Affiliated Hospital of Harbin Medical University, Harbin, Heilongjiang China; 3 The Department of Anesthesiology, the First Affiliated Hospital of Harbin Medical University, Harbin, Heilongjiang China; 4 The Department of Oncology and Vascular Interventional Radiology, Zhongshan Hospital, Xiamen University, Xiamen, China

**Keywords:** Weighted gene co-expression network analysis, Differentially expressed genes, Overall survival, Melanoma, *BRAF* gene

## Abstract

**Background:**

Primary cutaneous malignant melanoma is a cancer of the pigment cells of the skin, some of which are accompanied by *BRAF* mutation. Melanoma incidence and mortality rates have been rising around the world. As the current knowledge about pathogenesis, clinical and genetic features of cutaneous melanoma is not very clear, we aim to use bioinformatics to identify the potential key genes involved in the expression and mutation status of *BRAF*.

**Methods:**

Firstly, we used UCSC public hub datasets of melanoma (Lin et al., Cancer Res 68(3):664, 2008) to perform weighted genes co-expression network analysis (WGCNA) and differentially expressed genes analysis (DEGs), respectively. Secondly, overlapping genes between significant gene modules and DEGs were screened and validated at transcriptional levels and overall survival in TCGA and GTEx datasets. Lastly, the functional enrichment analysis was accomplished to find biological functions on the web-server database.

**Results:**

We performed weighted correlation network and differential expression analyses, using gene expression data in melanoma samples. We identified 20 genes whose expression was correlated with the mutation status of *BRAF*. For further validation, three of these genes (*CYR61*, *DUSP1*, and *RNASE4*) were found to have similar expression patterns in skin tumors from TCGA compared with normal skin samples from GTEx. We also found that weak expression of these three genes was associated with worse overall survival in the TCGA data. These three genes were involved in the nucleic acid metabolic process.

**Conclusion:**

In this study, *CYR61*, *DUSP1*, and *RNASE4* were identified as potential genes of interest for future molecular studies in melanoma, which would improve our understanding of its causes and underlying molecular events. These candidate genes may provide a promising avenue of future research for therapeutic targets in melanoma.

**Electronic supplementary material:**

The online version of this article (10.1186/s12881-019-0791-1) contains supplementary material, which is available to authorized users.

## Background

Skin cutaneous melanoma (SKCM) is a malignant cancer that originates from melanocytes and exists in different forms. The main types are basal cell cancer (BCC), squamous cell cancer (SCC) and melanoma [1, 2]. Melanoma is the most dangerous type of skin cancer. The primary cause of melanoma is ultraviolet light (UV) exposure in those with low levels of skin pigment [1, 2]. The UV light may come from the sun or other sources, such as artificial light devices. Besides, about 25 % of melanoma derives from moles. Those with many moles, a history of affected family members, and who have poor immune function were at greater risk [2]. A number of rare genetic defects such as xeroderma pigmentosum also increase risk [3]. Diagnosis can be finished by biopsy of any concerning skin lesion [2].

At least 50 % of melanomas harbor a V600E mutation in the *BRAF* gene. Tumors with *BRAF* mutations could respond to *BRAF* kinase inhibitor vemurafenib that was approved by the FDA in 2011 for therapy of patients with advanced melanoma and late-stage (metastatic) melanoma [4, 5]. Recently, the FDA approved the other two drugs named dabrafenib and ipilimumab as therapy for patients with BRAF V600E mutation-positive in melanoma [6].

Existing research has revealed that cancer cannot be caused by only one gene or factor. It must be a network of different genes and pathways working together. Weighted gene co-expression network analysis (WGCNA) [7] is a methodology used to analyze novel gene modules co-expressing in gene expression data. Many studies have shown that WGCNA can be used to explore genes, a network of genes and correlation of genes in different cancers [8, 9]. Moreover, differentially expressed genes (DEGs) analysis method has been applied in gene expression data [10].

In this paper, the study was designed to find potential genes and correlated pathways associated with the expression level and mutation status of *BRAF* in melanoma samples. By analyzing gene expression data [11] from UCSC public hub with the WGCNA algorithm and DEGs analysis, significant gene modules associated with the expression level of *BRAF* were identified and differentially expressed genes associated with the mutation status of *BRAF* were screened, then overlapping genes were validated in TCGA and GTEx database.

## Materials and methods

### Data collection

A dataset containing the gene expression and basic phenotypes information of 95 melanoma samples was downloaded from the Cancer Browser website (https:// xenabrowser.net/datapages/?cohort=Melanoma%20 (Lin%202,008)). The gene expression information was experimentally collected through GeneChip Fluidics Station (Affymetrix), and the matrix values were log_2_ ratio transformed. Genes were mapped onto Affymetrix HT-HGU133A probeMAP.

### Study population

Melanoma samples that had both expression data and *BRAF* mutation status were included for further analysis. According to this criterion, there were 67 melanoma samples (30 *BRAF* wild-type and 37 *BRAF* mutation) corresponding to our analysis requirement.

### Data processing

**Fig. 1:**
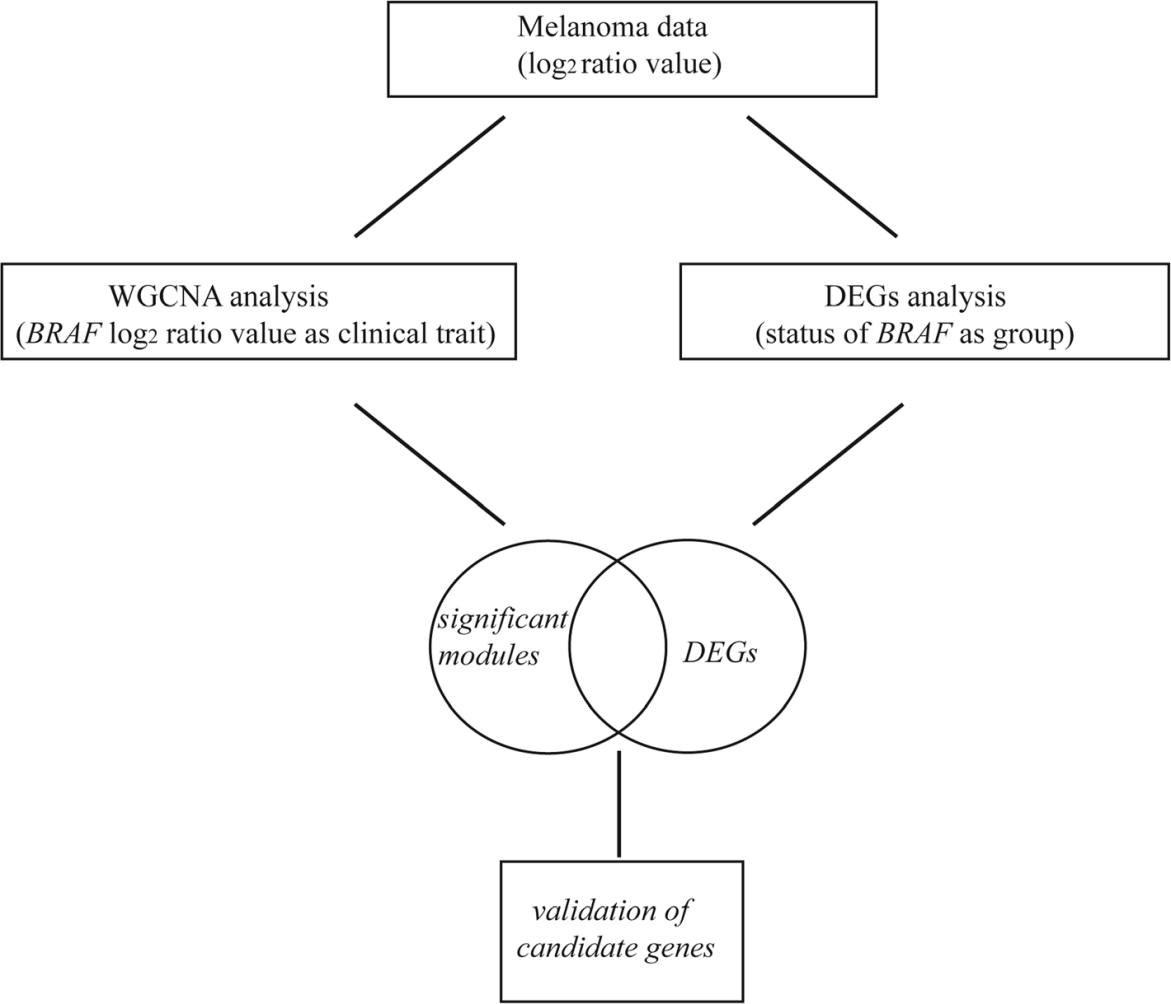
Data analysis workflow

After the dataset was downloaded, probe identification numbers (IDs) were transformed into gene symbols. For multiple probes corresponding to one gene, the probe with the most significant *p*-value from the downstream differential analysis was retained as the gene expression value. As for DEGs analysis, we divided 67 samples into two groups (*BRAF* wild-type and *BRAF* mutation group) for screening differentially expressed genes. As for WGCNA analysis, we used *BRAF* gene expression values as clinical trait data. Figure 1 shows the paths of the data analysis.

### Weighted gene co-expression network construction

The full set of genes with available expression data (10,994 genes) was applied to find the scale-free gene modules of co-expression and highly correlated genes constructed by WGCNA [7]. To construct a weighted gene network, the soft threshold power β was set to 3, which was the lowest power based on scale-free topology [12]. We set the parameter maxBlockSize = 11,000, and TOMType = “unsigned”. Topological overlap matrix (TOM) was calculated by adjacency transformation, and the value (1-TOM) was designated to the distance for identification of hierarchical clustering genes and modules. The minimum module size was set to 30.

### Module clinical feature associations

In order to identify modules that were significantly associated with the designated clinical trait (the expression level of *BRAF*), we plotted the heat map of modules-trait relationship according to the tutorial of the WGCNA package for R.

### Identification of DEGs

Linear models for microarray data (*limma* package) is a library used for analyzing gene expression microarray data [13], especially for the assessment of differential expression and the analysis of designed experiments [14, 15]. *limma* package in R has been applied to identify the DEGs between *BRAF* mutation and wild-type (marked as control group) samples. Genes with |log2 fold change (FC)| ≥ 1 and adjusted *p*-value < 0.05 as the cut-off criterion were selected for subsequent analysis.

### Validation of candidate genes

The overlapping genes between significant modules and DEGs were chosen as the potential genes for deep analysis and validation. GEPIA [16] (website: http://gepia.cancer-pku.cn/) is a web server for analyzing the RNA sequencing expression data of 9736 tumors and 8587 normal samples from the TCGA and the GTEx projects, using a standard processing pipeline. Survival analysis and expression consistency evaluation of potential genes were carried out in GEPIA built-in SKCM and GTEx datasets, which contain 461TCGA-SKCM tumor patients, 1TCGA-SKCM normal control, and 557 GTEx normal skin samples. For the transcriptional level validation, the criteria of significant results was set to |log2 fold change| ≥ 1 and *p*-value < 0.01. For the overall survival analysis in TCGA datasets, the 458 samples with available overall survival data were divided into high and low expression groups using the median TPM as a breakpoint, and significance was determined using a logrank test with *p* < 0.05.

### Functional enrichment analysis

GenCLiP 2.0 [17] is a web-based text-mining server, which can analyze human genes associated with biological functions and molecular networks. We uploaded filtered genes to online analysis tool GenCLiP 2.0 (http://ci.smu.edu.cn/GenCLiP2/ analysis.php) to find correlated significant pathways.

## Results

### Expression value analysis of microarray data

**Fig. 2:**
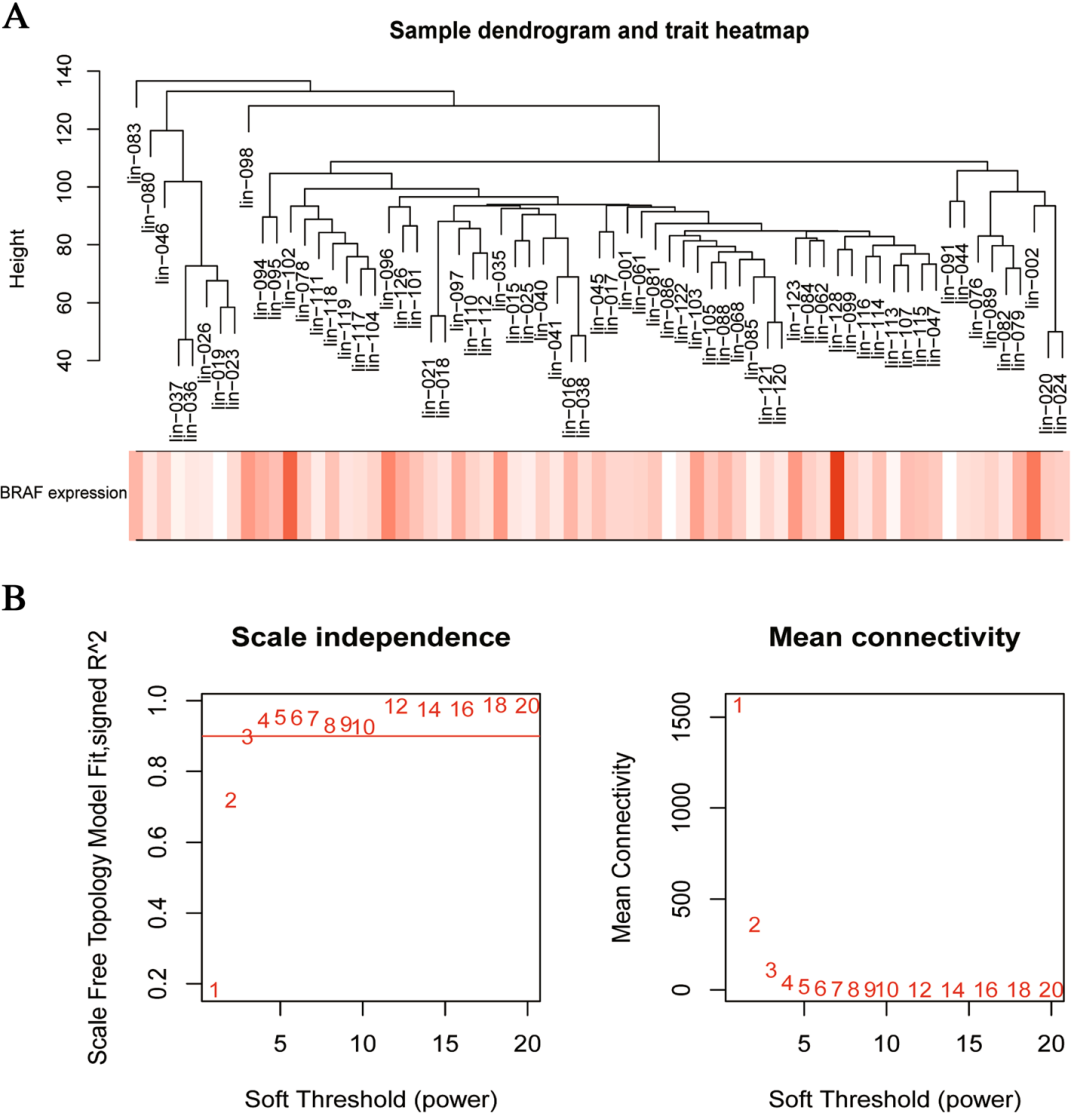
The clustering of samples and selection of soft-thresholding power. **a** The clustering dendrogram of samples based on their Euclidean distance. **b** Analysis of the scale-free fit index for various soft-thresholding powers

We chose 10,994 genes and 67 samples to construct the gene co-expression network by WGCNA. Figure 2a showed the relationship between the expression level of *BRAF* and melanoma samples.

### Weighted gene co-expression network construction

**Fig. 3:**
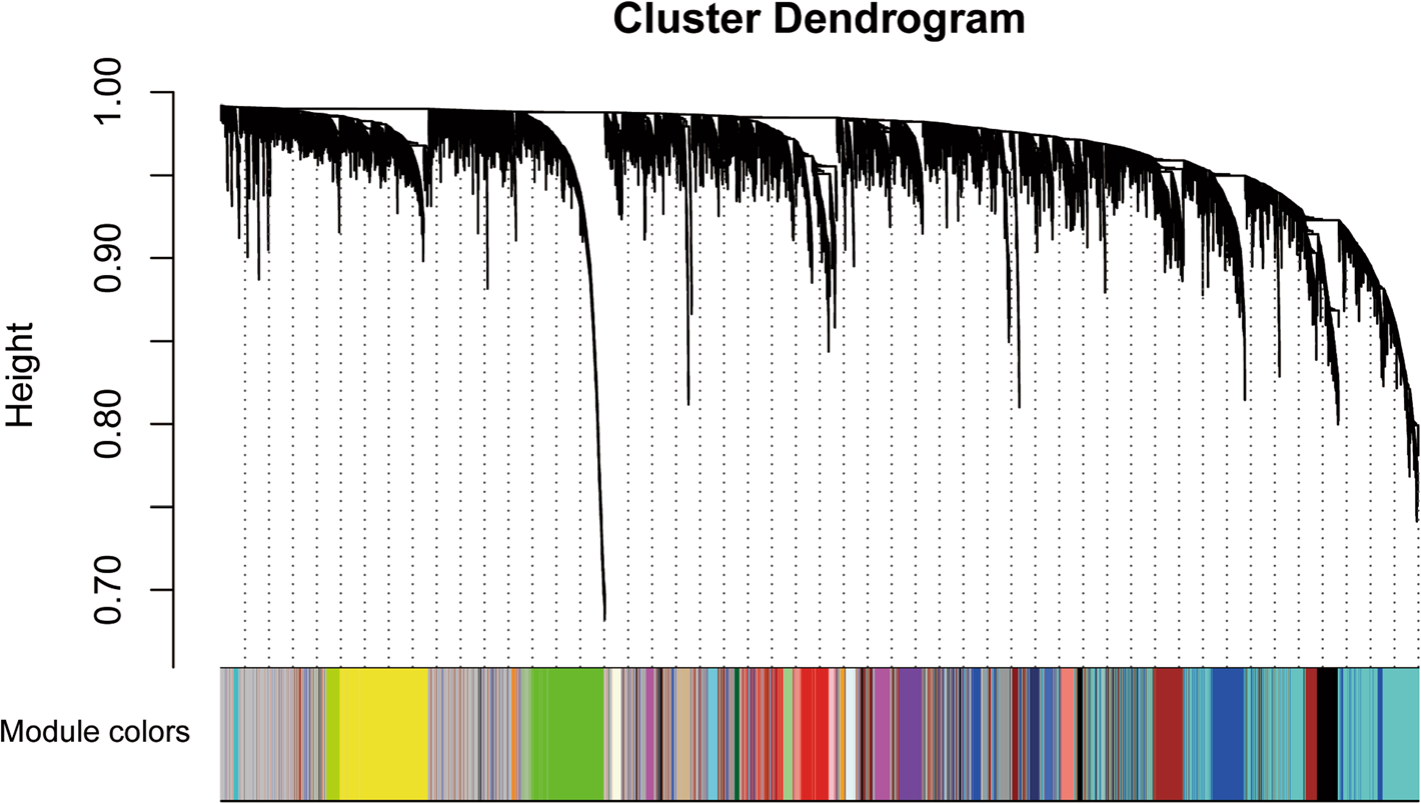
The clustering dendrogram of genes in melanoma, every color below represents one co-expression gene module

Choosing a proper soft-thresholding power is a critical step when constructing a WGCNA network. As shown in Fig. 2b, power value 3(β = 3) was selected to produce a hierarchical clustering tree (Fig. 3) with different colors representing different modules.

### Module clinical feature associations

**Fig. 4:**
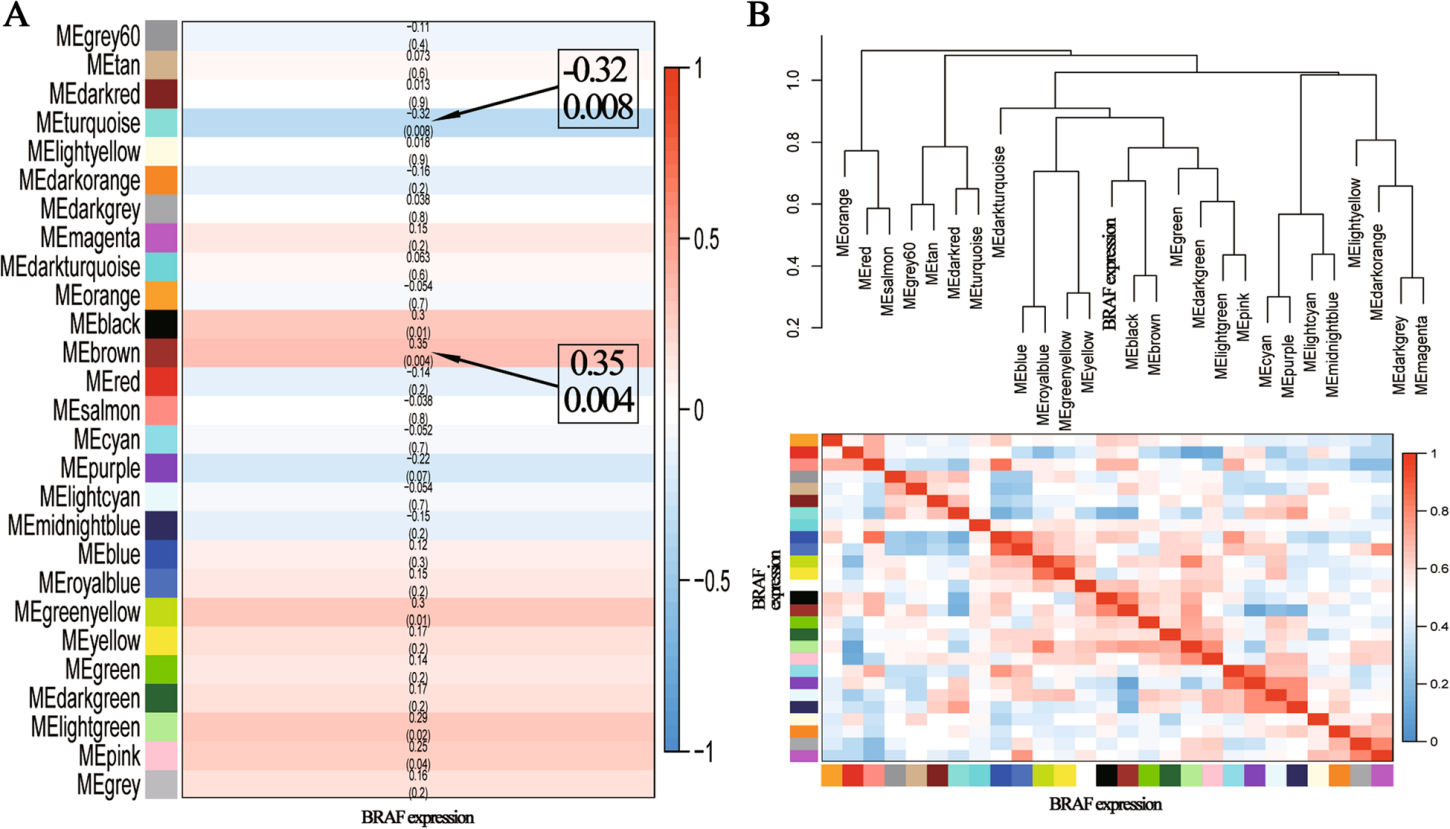
**a** Heatmap of module-trait relationships. The brown module was the most positive module (correlation coefficients: 0.35, and *p*-value: 0.004) correlated with the expression of *BRAF*, and the turquoise was the most negative module (correlation coefficients: −0.32, and *p*-value: 0.008). **b** Hierarchical clustering of module and heatmap plot of the eigengene adjacencies

Since we had a summary profile (eigengene) for each module, we simply correlated eigengenes with external traits (marked *BRAF* expression) and looked for the most significant associations. It was clear that the MEbrown (1021 genes) was most positive associated with the expression of *BRAF* (Fig. 4a). The results also demonstrated that the MEturquoise (1858 genes) was most negative associated with the expression of *BRAF* (Fig. 4a).

As shown in Fig. 4b, there were 27 eigengenes. The upper panels presented hierarchical clustering dendrograms of the eigengenes, in which the dissimilarity of eigengenes had been visualized. The bottle heatmaps presented the eigengene adjacencies for the expression of *BRAF*. The dendrogram indicated that brown and black modules were highly related and their correlations were stronger than their individual correlations with the expression *BRAF* (Fig. 4b).

### Identification of DEGs

Compared with *BRAF* wild type group, a total of 36 genes were identified in *BRAF* mutation group by the threshold of |log2 fold change (FC)| ≥ 1 and adjusted *p*-value < 0.05, of which 9 were up-regulated genes and 27 were down-regulated genes (Table 1).

**Table 1:** Thirty-six differentially expressed genes (DEGs) were identified from melanoma, including 9 up-regulated genes and 27 down-regulated genes. (The up-regulated genes were listed from the largest to the smallest of fold changes, and down-regulated genes were listed from the smallest to largest)

DEGs	Genes
Up-regulated	*MGP, ASB9, FCDH7, FXYD3, SORL1, PCSK6, MGST2, ITGB3, CORO2B*
Down-regulated	*MME, CYR61, CXCL1, MYL9, FOS, MICAL2, MFAP2, ID3, TXNIP, TNFAIP3, COX7A1, DUSP1, RNASE4, GALC, ANGPTL4, IFI6, NCRNA00312, FHOD3, ZSCAN18, PTEN, WSB1, SOD2, NID2, ANG, FERMT2, DACT1, FAM69A*

### Validation of candidate genes

**Fig. 5:**
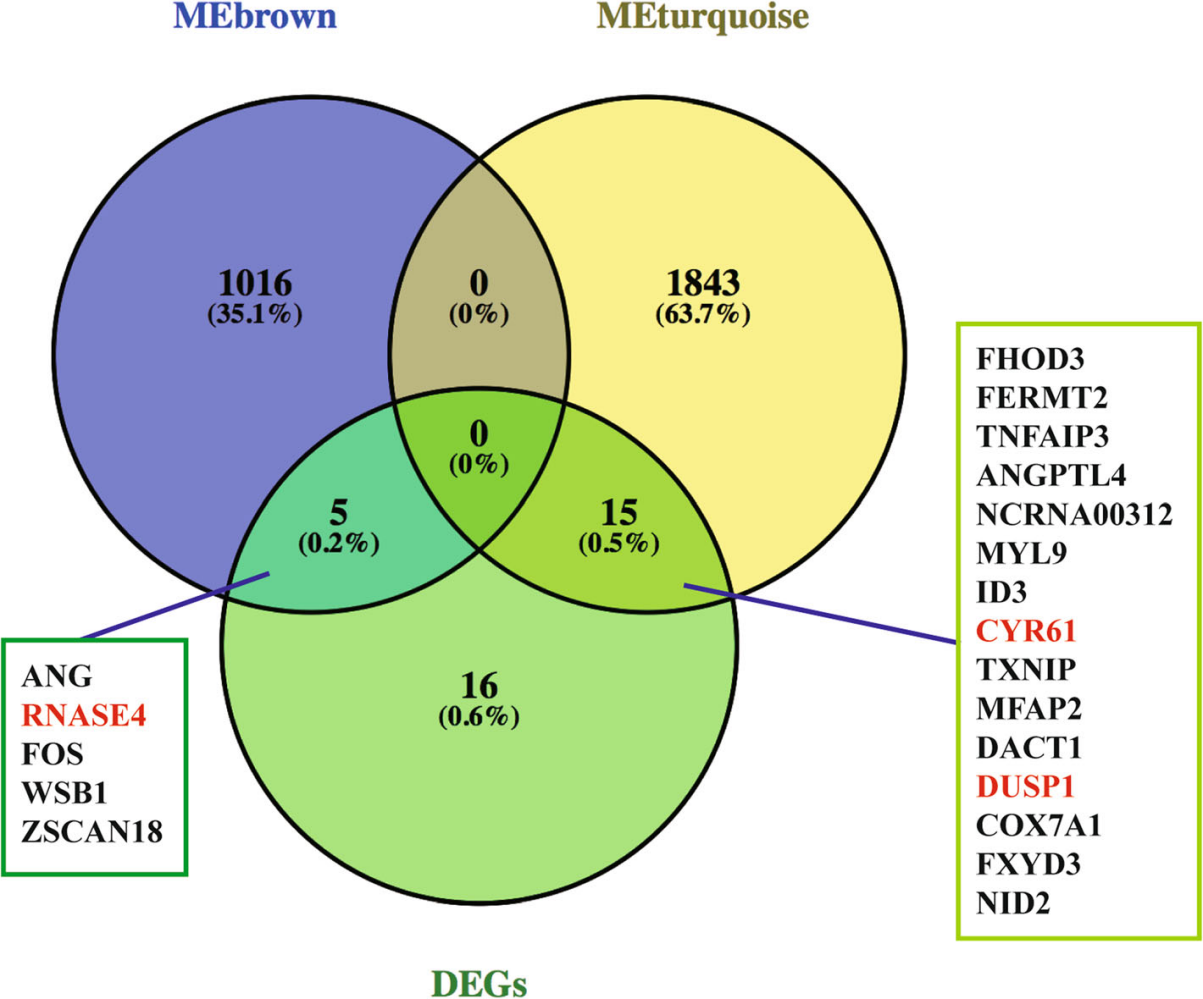
A Venn diagram showing the overlapping genes between modules and DEGs. Genes marked in red met the screening criteria and were chosen as the final set of candidate genes

There were 1021 genes in the brown module, 1858 genes in the turquoise module and 36 genes in the DEGs (Fig. 5). As shown in Venn diagram, it had 5 genes (*ANG, RNASE4, FOS, WSB1, ZSCAN18*) between MEbrown and DEGs, and 15 genes (*FHOD3, FERMT2, TNFAIP3, ANGPTL4, NCRNA00312, MYL9, ID3, CYR61, TXNIP, MFAP2, DACT1, DUSP1, COX7A1, FXYD3, NID2*) between MEturquoise and DEGs (Fig. 5).

**Fig. 6:**
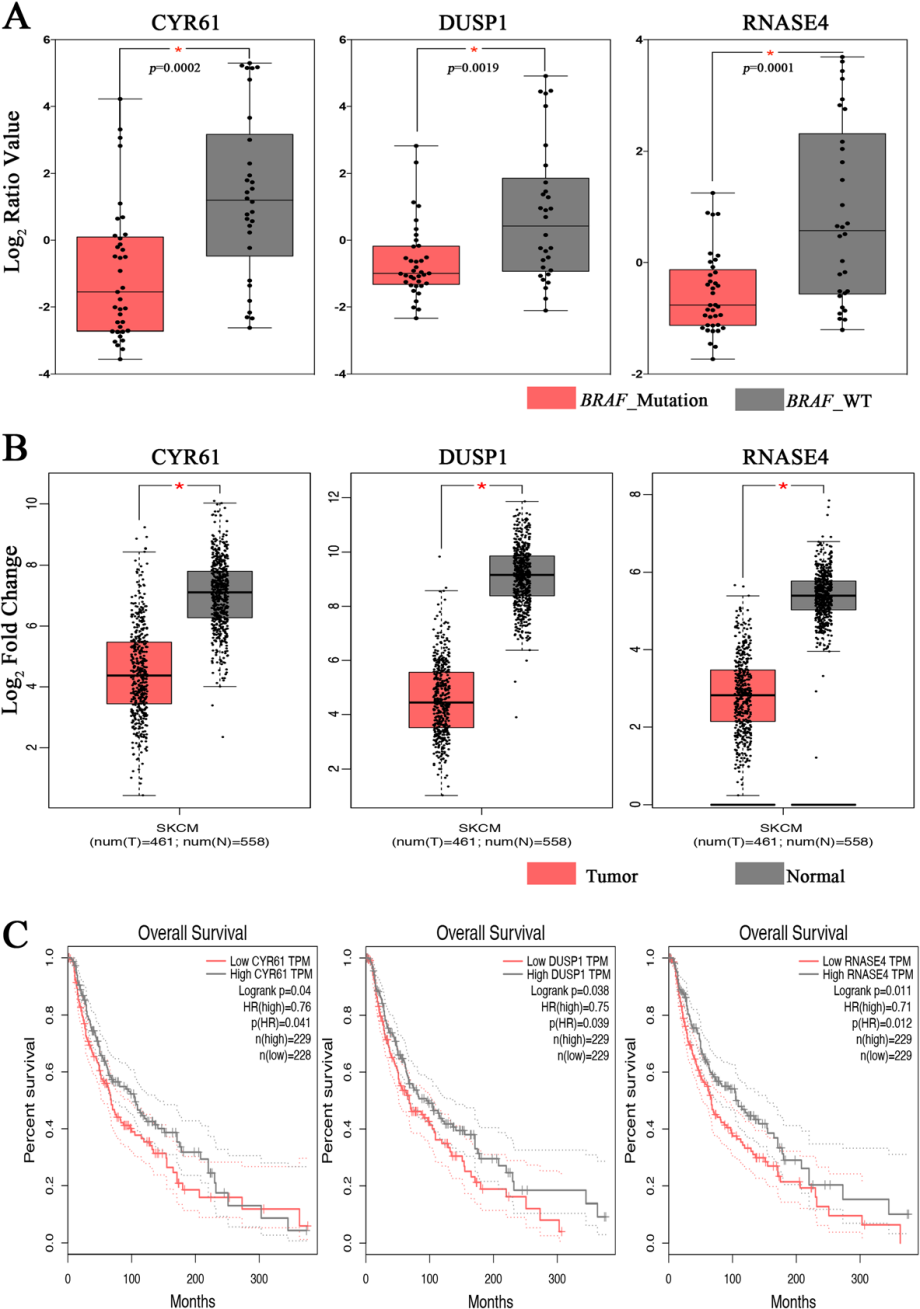
**a** The gene expression (log2 ratio value) of *CYR61*, *DUSP1*, and *RNASE4* in melanoma samples (unpaired t test, * indicates *p* < 0.01). **b** Validation of the gene expression of *CYR61*, *DUSP1*, and *RNASE4* in TCGA-SKCM (including 461 tumor patients and 1 normal control) and GTEx (including 557 normal control). The cutoff was set to |log2 fold change (FC)| ≥ 1, and *p* < 0.01. * indicates *p* < 0.01. **c** Overall survival analysis of the expression level of *CYR61*, *DUSP1*, and *RNASE4* in TCGA-SKCM on GEPIA website

In order to verify these 20 overlapping candidate genes, we validated on online web server GEPIA, which contained the TCGA and GTEx melanoma samples. Figure 6a-b demonstrated the expression level of 3 genes in *BRAF* wild-type and *BRAF* mutation samples of melanoma, which was in accordance with its expression level in normal and tumor patients of SKCM. It was also revealed that low expression of these three genes has a worse overall survival in SKCM patients (Fig. 6c). Besides, we had discarded the other 17 genes that did not exhibit significant differential expression in the TCGA/GTEx data concordant with that observed in the Lin et al. data, and were not associated with significantly worse overall survival compared high expression group with low expression group in the TCGA/GTEx data (Additional file 1: Figure S1, S2 and S3).

### Functional enrichment analysis

We used online website GenCLiP 2.0 tools to perform the functional and signaling pathway enrichment analysis of the above three genes (*CYR61, DUSP1,* and *RNASE4*). As shown in Table 2, the potential candidate genes (*CYR61, DUSP1,* and *RNASE4*) were involved in the nucleic acid metabolic process, while *CYR61* and *DUSP1* were most significantly enriched in the growth factor binding, *ERK1* and *ERK2* cascade, and regulation of *ERK1* and *ERK2* cascade.

**Table 2:** The gene ontology analysis of potential key genes in melanomas

ID	Term	Count	*p*-value	Genes
GO:0016788	hydrolase activity, acting on ester bonds	2	0.00465	*CYR61, RNASE4*
GO:0090304	nucleic acid metabolic process	3	0.02260	*CYR61, DUSP1, RNASE4*
GO:0008219	cell death	2	0.03509	*CYR61, DUSP1*
GO:0071495	cellular response to endogenous stimulus	2	0.01713	*CYR61, DUSP1*
GO:0071310	cellular response to organic substance	2	0.04895	*CYR61, DUSP1*
GO:0009790	embryo development	2	0.008327	*CYR61, DUSP1*
GO:0048598	embryonic morphogenesis	2	0.00303	*CYR61, DUSP1*
GO:0019838	growth factor binding	2	0.00014	*CYR61, DUSP1*
GO:0006915	apoptotic process	2	0.03095	*CYR61, DUSP1*
GO:0043066	negative regulation of apoptotic process	2	0.00673	*CYR61, DUSP1*
GO:0060548	negative regulation of cell death	2	0.007915	*CYR61, DUSP1*
GO:0043069	negative regulation of programmed cell death	2	0.00688	*CYR61, DUSP1*
GO:0048646	anatomical structure formation involved in morphogenesis	2	0.01169	*CYR61, DUSP1*
GO:0016310	phosphorylation	2	0.04169	*CYR61, DUSP1*
GO:0043065	positive regulation of apoptotic process	2	0.00311	*CYR61, DUSP1*
GO:0010942	positive regulation of cell death	2	0.00354	*CYR61, DUSP1*
GO:0043068	positive regulation of programmed cell death	2	0.00316	*CYR61, DUSP1*
GO:0012501	programmed cell death	2	0.03166	*CYR61, DUSP1*
GO:0006468	protein phosphorylation	2	0.02993	*CYR61, DUSP1*
GO:0006508	proteolysis	2	0.02311	*CYR61, DUSP1*
GO:0070372	regulation of ERK1 and ERK2 cascade	2	0.00054	*CYR61, DUSP1*
GO:0043408	regulation of MAPK cascade	2	0.00578	*CYR61, DUSP1*
GO:0042981	regulation of apoptotic process	2	0.01902	*CYR61, DUSP1*
GO:0050790	regulation of catalytic activity	2	0.04813	*CYR61, DUSP1*
GO:0010941	regulation of cell death	2	0.02167	*CYR61, DUSP1*
GO:0051128	regulation of cellular component organization	2	0.04404	*CYR61, DUSP1*
GO:1902531	regulation of intracellular signal transduction	2	0.02427	*CYR61, DUSP1*
GO:0043549	regulation of kinase activity	2	0.00783	*CYR61, DUSP1*
GO:0019220	regulation of phosphate metabolic process	2	0.02663	*CYR61, DUSP1*
GO:0051174	regulation of phosphorus metabolic process	2	0.02702	*CYR61, DUSP1*
GO:0042325	regulation of phosphorylation	2	0.02016	*CYR61, DUSP1*
GO:0043067	regulation of programmed cell death	2	0.01933	*CYR61, DUSP1*
GO:0070371	ERK1 and ERK2 cascade	2	0.00060	*CYR61, DUSP1*
GO:0000165	MAPK cascade	2	0.00687	*CYR61, DUSP1*
GO:0009888	tissue development	2	0.02669	*CYR61, DUSP1*
GO:0044702	single organism reproductive process	2	0.01329	*CYR61, DUSP1*
GO:0023014	signal transduction by protein phosphorylation	2	0.00735	*CYR61, DUSP1*
GO:0009719	response to endogenous stimulus	2	0.02830	*CYR61, DUSP1*
GO:0022414	reproductive process	2	0.01641	*CYR61, DUSP1*
GO:0000003	reproduction	2	0.01646	*CYR61, DUSP1*
GO:0051338	regulation of transferase activity	2	0.01001	*CYR61, DUSP1*
GO:0030162	regulation of proteolysis	2	0.00476	*CYR61, DUSP1*
GO:0001932	regulation of protein phosphorylation	2	0.01783	*CYR61, DUSP1*
GO:0031399	regulation of protein modification process	2	0.02839	*CYR61, DUSP1*
GO:0045859	regulation of protein kinase activity	2	0.00701	*CYR61, DUSP1*

## Discussion

Melanoma is the most fatal form of skin cancer and strikes tens of thousands of people worldwide each year. The amount of cases is increasing faster than any other type of malignant cancer [18].

Many patients with *BRAF* mutation have received target treatments and therapies which activate their body’s own immune system. There is *BRAF* mutation in melanoma. Besides, mutation also exists in *NRAS* gene and *PTEN* gene. Some scientists have struggled to find drugs targeting the mutated *NRAS* protein or *NRAS* protein [19], while others have uncovered a mechanism of resistance of targeted therapies for melanoma and identified compounds that inhibit *eIF4F* and enhance the effectiveness of vemurafenib in mice with melanomas [20].

In this study, firstly we applied WGCNA to identify the two key modules in melanoma that were associated with the expression of *BRAF* gene (the brown module was positive, and the turquoise was negative). At the same time, we identified the DEGs in the *BRAF* mutation group compared with *BRAF* wild-type group. Then, we chose the overlapping genes between modules and DEGs. Finally, as to the gene expression level and overall survival validation, we expand the scope of comparison range to the tumor group versus the normal group in TCGA/GTEx datasets.

We found that *CYR61, DUSP1,* and *RNASE4* were significantly related to gene expression level and survival analysis results. *CYR61* (Cysteine-rich angiogenic inducer 61) is a secreted, matricellular protein [21], which is associated with a range of cellular activities, such as cell adhesion, migration, differentiation, proliferation, apoptosis [21, 22]. Beak et al. suggested that *CYR61* was highly expressed in colorectal carcinomas (CRC) and *CYR61* might play a role as meaningful targets for therapeutic intervention of patients with CRC [23]. D’ Antonio et al. also found that decreased expression level of *CRY61* was associated with prostate cancer recurrence after surgical treatment [24]. *DUSP1* (Dual specificity protein phosphatase 1) is an oncogene that is associated with cancer progression in gastric cancer as well as a negative regulator of the mitogen-activated protein kinase (MAPK) signaling pathway, has anti-inflammatory properties [25–27]. Xiaoyi et al. also found that *DUSP1* phosphatase regulated the pro-inflammatory milieu in head and neck squamous cell carcinoma [28], in addition to promoting angiogenesis, invasion, and metastasis in non-small-cell lung cancer (NSCLC) [29]. *RNASE4* (Ribonuclease 4) is an RNase that belongs to the pancreatic ribonuclease family and has marked specificity towards the 3′ side of uridine nucleotides [30]. Unfortunately, to date there has been no research focused on the relationship between these several genes with melanoma.

The primary purpose of the study focuses on the prediction of key potential genes in cancers via data mining and data analysis. Though we have validated results in the TCGA and GTEx datasets, results need to be confirmed through molecular and cellular experiments.

## Conclusions

Firstly, we have identified overlapping genes associated with the expression and the mutation status of *BRAF* in melanoma through WGCNA and DEGs analysis, respectively. Then, validation was applied to these overlapping genes, and three genes (*CYR61*, *DUSP1*, and *RNASE4*) were screened. However, more direct evidence is needed to confirm their association with melanoma. The study may be helpful for future studies concerning melanoma with the aim of finding potential key molecule targets of melanoma.

## Additional file

Additional file 1: **Figures S1-S3.** Rows represent expression of 17 genes in melanoma samples (first), TCGA/GTEx (second), and TCGA (third), where genes were aligned by column. As the NCRNA00312 gene could not be retrieved, expression and survival results could not be obtained in GEPIA. Significance was determined as described in the caption of Fig. 6. (ZIP 3130 kb)

## Abbreviations

BRAF: B-Raf proto-oncogene, serine/threonine kinase
DEGs: Differentially Expressed Genes
GO: Gene Ontology
UCSC: University of California Santa Cruz
WGCNA: Weighted Gene Co-expression Network Analysis

## References

[1] Stewart B, Wild C (2014). World cancer report 2014: International Agency for Research on Cancer.

[2] Version LCPP, Prevention LC. Skin Cancer Prevention (PDQ®) - National Library of Medicine - PubMed Health. National Cancer Institute 2013. https://www.cancer.gov/types/skin/hp/skin-prevention-pdq.

[3] Azoury SC, Lange JR (2014). Epidemiology, risk factors, Prevention, and early detection of melanoma. Surg Clin North Am.

[4] Reports FS (2011). FDA approves vemurafenib and companion diagnostic test for late-stage skin cancer.

[5] Sponghini AP, Rondonotti D, Giavarra M, Giorgione R, Platini F (2015). Safety and efficacy of vemurafenib in BRAF V600E mutation-positive metastatic melanomas. J Transl Med.

[6] Shahabi V, Hamid O, Schmidt H, Chasalow SD, Alaparthy S, Jackson JR (2012). Assessment of association between BRAF-V600E mutation status in melanomas and clinical response to ipilimumab. Cancer Immunol Immunother.

[7] Langfelder P, Horvath S. WGCNA: an R package for weighted correlation network analysis. BMC Bioinformatics. 2008;9(1):559. 10.1186/1471-2105-9-559 PMC2631488 19114008

[8] Yang Q, Wang R, Wei B, Peng C, Wang L, Hu G, Kong D, Du C. Candidate biomarkers and molecular mechanism investigation for glioblastoma Multiforme utilizing WGCNA. Biomed Res Int. 2018;2018. 10.1155/2018/4246703 PMC6178162 30356407

[9] Zhai X, Xue Q, Liu Q, Guo Y, Chen Z (2017). Colon cancer recurrence-associated genes revealed by WGCNA co-expression network analysis. Mol Med Rep.

[10] Rau A, Flister MJ, Rui H, Livermore Auer P. Exploring Drivers of Gene Expression in The Cancer Genome Atlas. 2018:227926. 10.1093/bioinformatics/bty551 PMC6298045 30561551

[11] Lin WM, Baker AC, Beroukhim R, Winckler W, Feng W, Marmion JM, Laine E, Greulich H, Tseng H, Gates C (2008). Modeling genomic diversity and tumor dependency in malignant melanoma. Cancer Res.

[12] Zhang B, Horvath S (2005). A General Framework For Weighted Gene Co-Expression Network Analysis. Stat Appl Genet Mol Biol.

[13] Chen-An T, Yi-Ju C, Chen JJ (2003). Testing for differentially expressed genes with microarray data. Nucleic Acids Res.

[14] Phipson B, Lee S, Majewski IJ, Alexander WS, Smyth GK (2016). Robust hyperparameter estimation protects against hypervariable genes and improves power to detect differential expression. Ann Appl Stat.

[15] Smyth GK. Limma: linear models for microarray data[M]//Bioinformatics and computational biology solutions using R and Bioconductor. New York: Springer; 2005. p. 397-420.

[16] Tang Z, Li C, Kang B, Gao G, Li C, Zhang Z. GEPIA: a web server for cancer and normal gene expression profiling and interactive analyses. Nucleic Acids Res. 2017;45 Web Server issue. 10.1093/nar/gkx247 PMC5570223 28407145

[17] Wang JH, Zhao LF, Lin P, Su XR, Chen SJ, Huang LQ, Wang HF, Zhang H, Hu ZF, Yao KT (2014). GenCLiP 2.0: a web server for functional clustering of genes and construction of molecular networks based on free terms. Bioinformatics.

[18] Owens B (2014). Melanoma. Nature.

[19] Posch C, Moslehi H, Feeney L, Green GA, Ebaee A, Feichtenschlager V, Chong K, Peng L, Dimon MT, Phillips T (2013). Combined targeting of MEK and PI3K/mTOR effector pathways is necessary to effectively inhibit NRAS mutant melanoma in vitro and in vivo. Proc Natl Acad Sci U S A.

[20] Boussemart L, Malka-Mahieu H, Girault I, Allard D, Hemmingsson O, Tomasic G, Thomas M, Basmadjian C, Ribeiro N, Thuaud F (2014). eIF4F is a nexus of resistance to anti-BRAF and anti-MEK cancer therapies. Nature.

[21] Lau LF (2011). CCN1/CYR61: the very model of a modern matricellular protein. Cell Mol Life Sci.

[22] Jun JI, Lau LF (2011). Taking aim at the extracellular matrix: CCN proteins as emerging therapeutic targets. Nat Rev Drug Discov.

[23] Baek M, Bae S, Jeong D (2011). Relationship of pro-angiogenic factor Cyr61to colorectal cancer development and prognosis, vol. 29.

[24] D'Antonio KB, Lucianna S, Roula A, Mondul AM, Platz EA, Netto GJ, Getzenberg RH (2010). Decreased expression of Cyr61 is associated with prostate cancer recurrence after surgical treatment. Clin Cancer Res.

[25] Peng HZ, Yun Z, Wang W, Ma BA (2017). Dual specificity phosphatase 1 has a protective role in osteoarthritis fibroblastlike synoviocytes via inhibition of the MAPK signaling pathway. Mol Med Rep.

[26] Teng F, Xu Z, Chen J, Zheng G, Zheng G, Lv H, Wang Y, Wang L, Cheng X (2018). DUSP1 induces apatinib resistance by activating the MAPK pathway in gastric cancer. Oncol Rep.

[27] Keyse SM, Emslie EA (1992). Oxidative stress and heat shock induce a human gene encoding a protein-tyrosine phosphatase. Nature.

[28] Xiaoyi Z, J Madison H, Hong Y, D'Silva NJ, Kirkwood KL: DUSP1 phosphatase regulates the proinflammatory milieu in head and neck squamous cell carcinoma. Cancer Res 2014, 74(24):7191–7197. 10.1158/0008-5472.CAN-14-1379 PMC4268021 25312268

[29] Moncho-Amor V, Cáceres IIED, Bandres E, Martínez-Poveda B, Orgaz JL, Sánchez-Pérez I, Zazo S, Rovira A, Albanell J, Jiménez B (2011). DUSP1/MKP1 promotes angiogenesis, invasion and metastasis in non-small-cell lung cancer. Oncogene.

[30] Rosenberg HF, Dyer KD (1995). Human ribonuclease 4 (RNase 4): coding sequence, chromosomal localization and identification of two distinct transcripts in human somatic tissues. Nucleic Acids Res.

