# Supplementary figures and images for: Weighted correlation network and differential expression analyses identify candidate genes associated with BRAF gene in melanoma
Source: BMC Med Genet. 2019 Mar 29;20:54. doi: 10.1186/s12881-019-0791-1 (PMC6441238; doi:10.1186/s12881-019-0791-1)

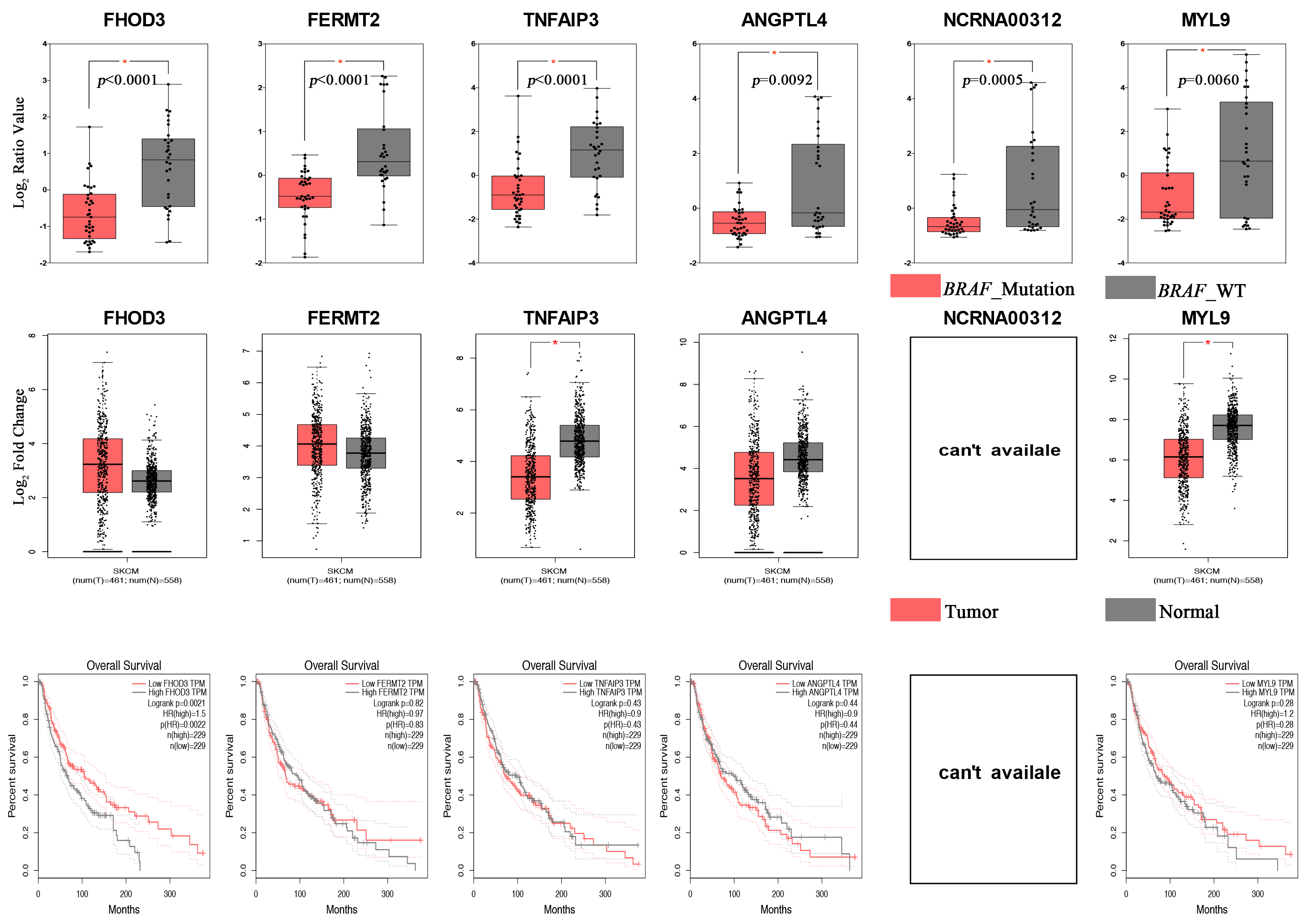

Supplement: Supplementary file 1 — Figures S1-S3. Rows represent expression of 17 genes in melanoma samples (first), TCGA/GTEx (second), and TCGA (third), where genes were aligned by column. As the NCRNA00312 gene could not be retrieved, expression and survival results could not be obtained in GEPIA. Significance was determined as described in the caption of Fig. 6. (ZIP 3130 kb) [file 12881_2019_791_MOESM1_ESM.zip › Figure S-1R4.tif]

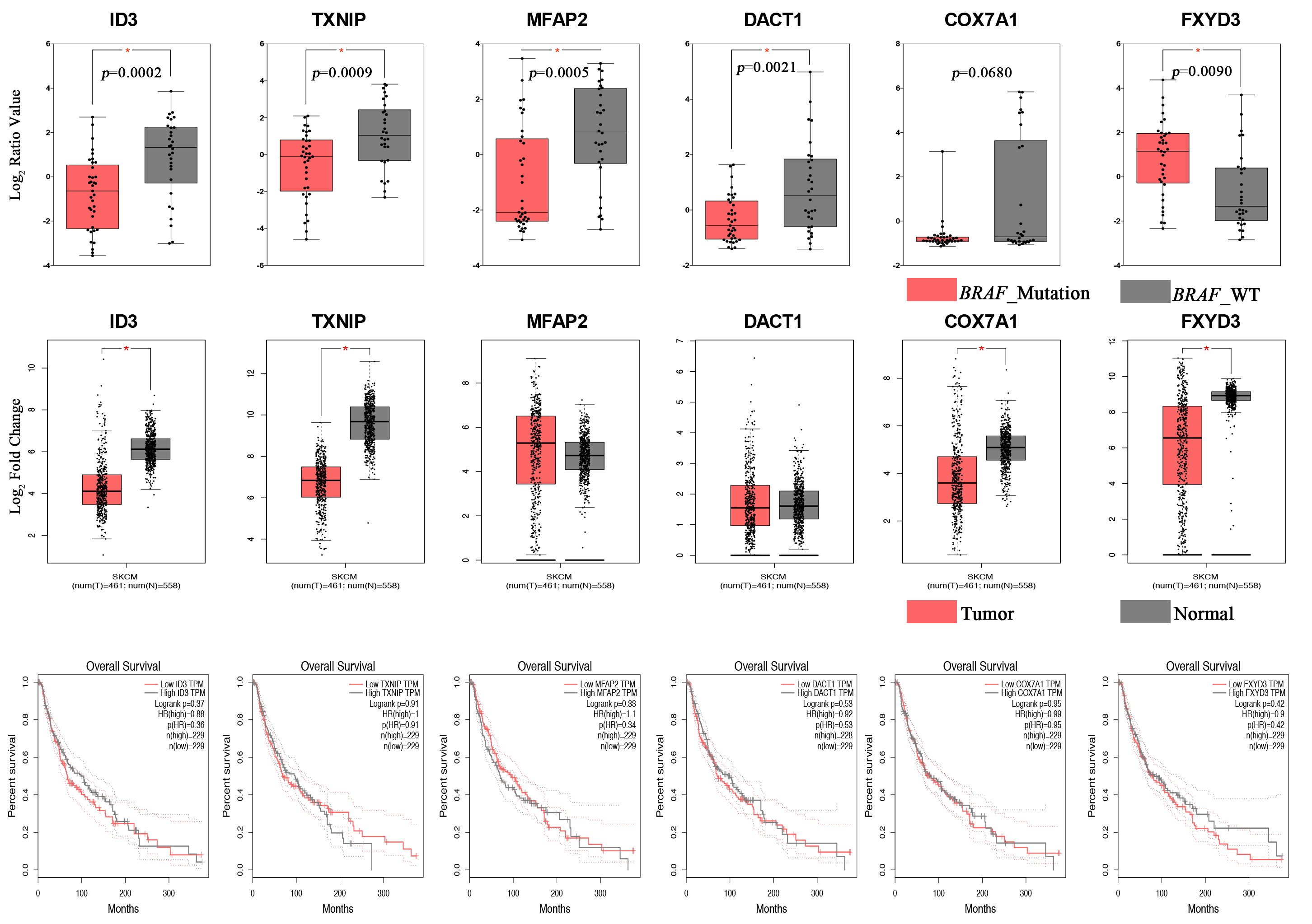

Supplement: Supplementary file 1 — Figures S1-S3. Rows represent expression of 17 genes in melanoma samples (first), TCGA/GTEx (second), and TCGA (third), where genes were aligned by column. As the NCRNA00312 gene could not be retrieved, expression and survival results could not be obtained in GEPIA. Significance was determined as described in the caption of Fig. 6. (ZIP 3130 kb) [file 12881_2019_791_MOESM1_ESM.zip › Figure S-2R4.tif]

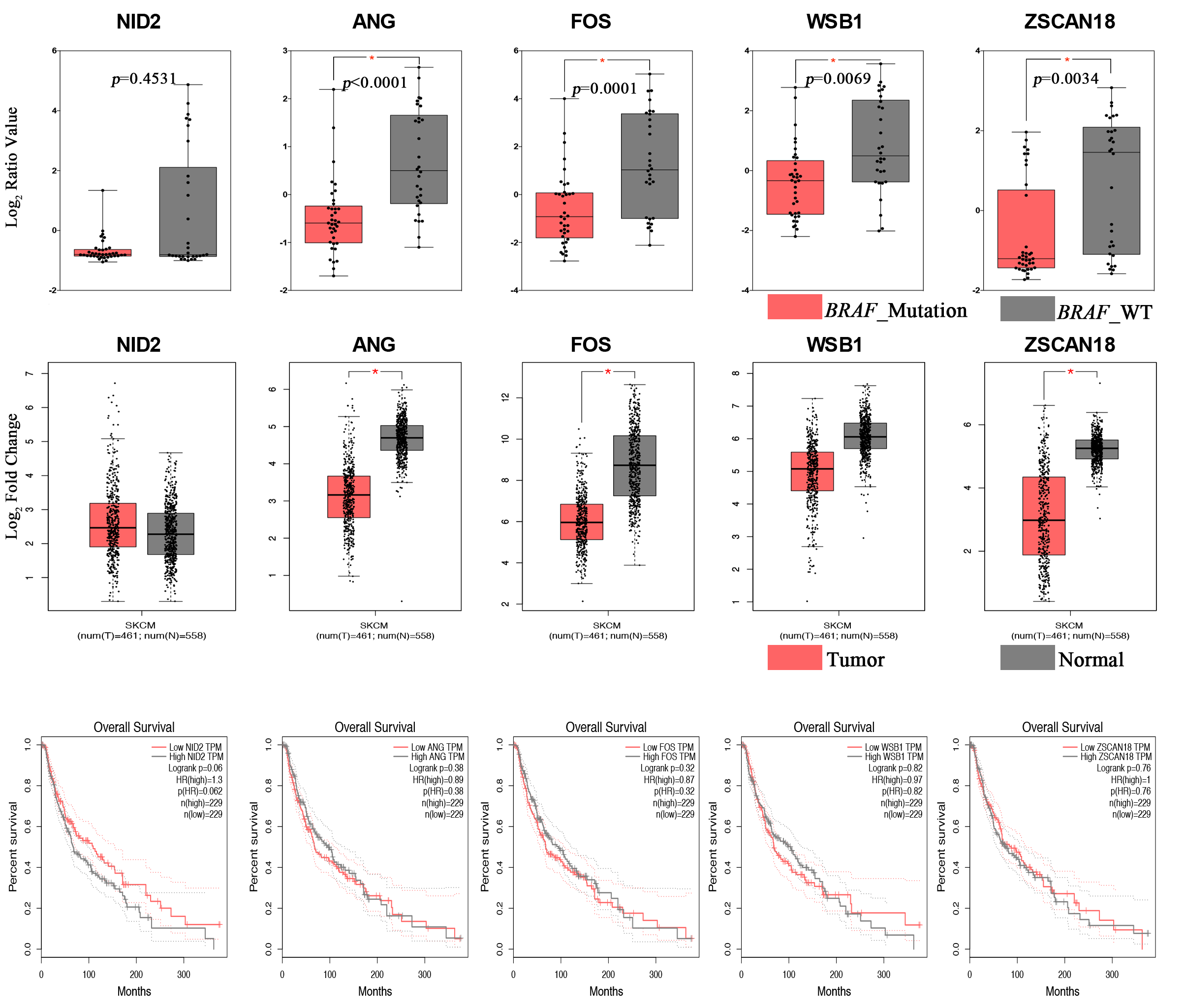

Supplement: Supplementary file 1 — Figures S1-S3. Rows represent expression of 17 genes in melanoma samples (first), TCGA/GTEx (second), and TCGA (third), where genes were aligned by column. As the NCRNA00312 gene could not be retrieved, expression and survival results could not be obtained in GEPIA. Significance was determined as described in the caption of Fig. 6. (ZIP 3130 kb) [file 12881_2019_791_MOESM1_ESM.zip › Figure S-3R4.tif]
